# Genome Sequence of a Thermoacidophilic Methanotroph Belonging to the Verrucomicrobiota Phylum from Geothermal Hot Springs in Yellowstone National Park: A Metagenomic Assembly and Reconstruction

**DOI:** 10.3390/microorganisms10010142

**Published:** 2022-01-11

**Authors:** Hye Won Kim, Na Kyung Kim, Alex P. R. Phillips, David A. Parker, Ping Liu, Rachel J. Whitaker, Christopher V. Rao, Roderick Ian Mackie

**Affiliations:** 1 Department of Animal Sciences, University of Illinois at Urbana-Champaign, Urbana, IL 61801, USA; wony9182@illinois.edu (H.W.K.); nkwellnaci627@gmail.com (N.K.K.); 2 Materials Research Laboratory, Energy & Biosciences Institute, University of Illinois at Urbana-Champaign, Urbana, IL 61801, USA; David.A.Parker@shell.com (D.A.P.); ping.liu@shell.com (P.L.); cvrao@illinois.edu (C.V.R.); 3 Department of Microbiology, University of Illinois at Urbana-Champaign, Urbana, IL 61801, USA; apphill2@illinois.edu (A.P.R.P.); rwhitakr@illinois.edu (R.J.W.); 4 Shell Exploration and Production Inc., Westhollow Technology Center, Houston, TX 77082-3101, USA; 5 Carl R. Woese Institute for Genomic Biology, University of Illinois at Urbana-Champaign, Urbana, IL 61801, USA; 6 Department of Chemical and Biomolecular Engineering, University of Illinois at Urbana-Champaign, Urbana, IL 61801, USA

**Keywords:** methanotroph, verrucomicrobiota, metagenome assembled genome, functional metabolism

## Abstract

Verrucomicrobiotal methanotrophs are thermoacidophilic methane oxidizers that have been isolated from volcanic and geothermal regions of the world. We used a metagenomic approach that entailed obtaining the whole genome sequence of a verrucomicrobiotal methanotroph from a microbial consortium enriched from samples obtained from Nymph Lake (89.9 °C, pH 2.73) in Yellowstone National Park in the USA. To identify and reconstruct the verrucomicrobiotal genome from Illumina NovaSeq 6000 sequencing data, we constructed a bioinformatic pipeline with various combinations of de novo assembly, alignment, and binning algorithms. Based on the marker gene (*pmoA*), we identified and assembled the *Candidatus Methylacidiphilum* sp. YNP IV genome (2.47 Mbp, 2392 ORF, and 41.26% GC content). In a comparison of average nucleotide identity between Ca. *Methylacidiphilum* sp. YNP IV and Ca. *Methylacidiphilum fumariolicum* SolV, its closest 16S rRNA gene sequence relative, is lower than 95%, suggesting that Ca. *Methylacidiphilum* sp. YNP IV can be regarded as a different species. The Ca. *Methylacidiphilum* sp. YNP IV genome assembly showed most of the key genes for methane metabolism, the CBB pathway for CO_2_ fixation, nitrogen fixation and assimilation, hydrogenases, and rare earth elements transporter, as well as defense mechanisms. The assembly and reconstruction of a thermoacidophilic methanotroph belonging to the Verrucomicrobiota phylum from a geothermal environment adds further evidence and knowledge concerning the diversity of biological methane oxidation and on the adaptation of this geochemically relevant reaction in extreme environments.

## 1. Introduction

Methanotrophs are a distinct group of bacteria that are able to grow on methane as their sole carbon and energy source [1]. They play an important role in the global carbon cycle, where they act as a methane sink for this greenhouse gas produced biologically and geothermally [2]. Significant amounts of geological methane are produced within the Earth’s crust, and the verrucomicrobiotal methanotrophs contribute to carbon cycling in these extreme environments [3]. Early studies resulted in the classification of methanotrophs into two major groups: Gammaproteobacteria and Alphaproteobacteria [4,5,6,7]. However, the diversity of aerobic methanotrophs was expanded by the identification and isolation of Verrucomicrobial methanotrophs [3,8,9,10,11], which have recently been assigned the valid phylum name Verrucomicrobiota [12]. A remarkable property of these methanotrophs is their ability to oxidize methane under the extreme pH and temperatures characteristic of volcanic or geothermal environments [13,14].

In the late 2000s, three independent studies reported the existence of verrucomicrobiotal methanotrophs that were distinct species of the genus *Candidatus Methylacidiphilum* in the phylum Verrucomicrobiota. The representative strains are Ca. *Methylacidiphilum fumariolicum* SolV, isolated from volcanic mudpots near Italy Naples in 2007, Ca. *Methylacidiphilum kamchatkense* Kam1, isolated from an acidic geothermal Kamchatka in Russia in 2008, and Ca. *Methylacidiphilum infernorum* V4, isolated from sediment from Hell’s Gate in New Zealand in 2007 [3,8,9]. Three mesophilic acidophilic verrucomicrobiotal methanotrophs, proposed as a *Methylacidimicrobium* genus, were also isolated from a volcanic soil in Italy [15]. In a recent study, six new Verrucomicrobiota methanotrophic isolates with strain names Fur, Rib, and Fdl, isolated from the Azores, Yel, isolated from United States, Ice, isolated from Iceland, and Phi, isolated from the Philippines, were reported [16]. All of these isolates are extremely thermophilic and acidophilic methanotrophs adapted to the highly acidic (pH 1–4) and high temperatures (up to 65 °C) characteristic of volcanic and geothermal environments. The recent addition to the cultured diversity of the group is *Methylacidimicrobium thermophilum* AP8, isolated from volcanic soil on Pantelleria island in Italy [17]. Two other closely related *Methylacidiphilum*, sp. IT5 and IT6, were also isolated from ponds at the Pisciarelli hot spring in Italy [18].

The draft genome of *M. fumariolicum* SolV and the complete genome sequence of *M. infernorum* V4 showed over 98% 16S rRNA gene sequence identity [11]; however, an average nucleotide identity (ANI) comparison revealed that *M. fumariolicum* SolV and *M. infernorum* V4 were two different species. The 16S rRNA gene of *M. kamchatkense* Kam1 and *M. fumariolicum* SolV was also 99.7% identical. The ANI comparison including *M. kamchatkense* Kam1 and *M. fumariolicum* SolV was calculated as 92.38 to 92.54% [19]. Like the three reported strains above, the six new isolates reported by Erikstad et al. (2019) showed more than 98.6% of 16S rRNA sequence identity with *M. fumariolicum* SolV, *M. infernorum* V4, and *M. kamchatkense* Kam1 [8]. Based on genome homology, however, they concluded that two new isolates, Yel from Yellowstone National Park in the USA and Phi from Makiling mud spring in the Philippines, constituted the novel Ca. *Methylacidiphilum* species [16].

The discovery of the genus Ca. *Methylacidiphilum* revealed the diverse metabolic pathways used by the verrucomicrobiotal methanotrophs, providing a comprehensive understanding of how they are able to grow and survive in extreme environments. Methane monooxygenase (MMO) is the key enzyme involved in methane metabolism, converting methane into methanol. It exists in two distinct forms: a membrane-associated particulate form (pMMO) and a soluble cytoplasmic form (sMMO) [20,21]. Ca. *Methylacidiphilum* genomes are known to contain operons encoding genes for pMMO but not sMMO [11,22]. Importantly, they contain XoxF-type methanol dehydrogenases, which contain the rare earth elements (REE) lanthanides together with the PQQ co-factor [23]. While the mechanism of lanthanide uptake is not fully understood, a TonB-dependent receptor is known to be required for the growth of the methylotrophic bacterium in the presence of REEs [24,25]. Utilization of hydrogen gas is also important for verrucomicrobiotal methanotrophs to persist and grow in the extreme environments. *M. fumariolicum* SolV was found to contain genes encoding two NiFe-hydrogenases (group 1d and 1h/5) [2] that channel electrons from hydrogen into the quinone pool for energy production [26]. Otherwise, *M. infernorum* RTK17.1 contain genes encoding two NiFe-hydrogenases (group 1d and 3b) [27].

Even though methanotrophs have been studied for many years, an isolation and genome analysis of verrucomicrobiotal methanotrophs is still limited as compared to the gammaproteobacterial and the alphaproteobacterial methanotrophs. The aim of this study was to reveal genomic characteristics of verrucomicrobiotal methanotrophs isolated from the acidic hot springs in Yellowstone National Park in the USA. We used a metagenomic approach to recover a verrucomicrobiotal genome from enrichment cultures of sediment samples showing methane consumption.

## 2. Materials and Methods

### 2.1. Sampling and Cultivation

Samples used in this study were collected from Nymph Lake, Norris Geyser Basin, and Mud Volcano in Yellowstone National Park (Step 1 in Figure 1A) in September 2017 under permit YELL-2017-SCI-5684. Samples of sediment plus spring water were collected from the sites as previously described [28]. In brief, water and sediment designated for culturing were collected in sterile bottles rinsed once with spring water and then kept at room temperature while being transported to the laboratory at the University of Illinois in Urbana–Champaign.

**Figure 1:**
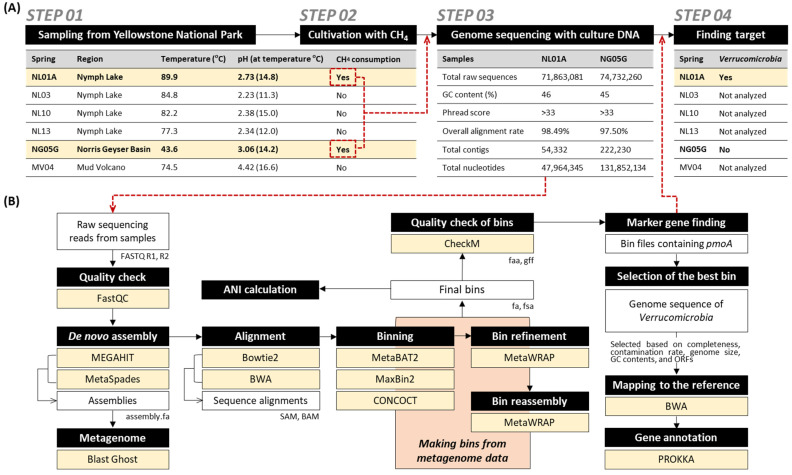
Overall process of metagenomic assembly and reconstruction of a verrucomicrobiotal genome from geothermal hot springs in Yellowstone National Park. (**A**) Steps from the environmental sampling to the finding target verrucomicrobiotal genome. (**B**) Computational pipeline for metagenomic data analysis and finding verrucomicrobiotal genome sequence in this study.

Samples (total 5 g of sediment plus spring water) were inoculated into the 40 mL of V42 mineral medium with the addition of REE trace supplement (0.2 µM of lanthanum and cerium) at pH 2.0 [8,27] and incubated at 60 °C with shaking (150 rpm) in triplicate. The headspace composition of the 120 mL serum bottles was 25% CH_4_ and 8% CO_2_ with the balance of air (total 80 mL of gas, 2 atm). After three serial transfers to enrich for methanotrophs, we confirmed that the enrichment culture of NL01A and NG05G showed methane consumption in headspace gas by gas chromatography (Gow-Mac series 580, GGOW-MAC Instrument Company, Bridgewater, NJ, USA) (Step 2 in Figure 1A). The cultures showing methane consumption were then serially diluted 10-fold with Phosphate Buffered Solution (PBS) and 100 µL of the dilutions were plated onto the V42 mineral salts gel agar (pH adjusted to 3.5 by H_2_SO_4_) containing 15 g/L of phytagel. Plates were incubated at 60 °C in anaerobic jars (Oxoid, UK) with the same gas composition described above, and picked colonies were identified by 16S rRNA gene sequencing.

### 2.2. 16S rRNA Gene Sequencing and Phylogenetic Analysis

Enriched cultures showing methane consumption as well as the picked colonies grown on the V42 plates were identified by 16S rRNA gene sequencing using the following primers: Verrucomicrobiota, Ver53F TGGCGGCGTGGATAAGA, 1492R GGCTACCTTGTTACGACTT [29]. Forward and reverse reads from the amplicons were analyzed with the National Center for Biotechnology Information (NCBI) nucleotide collection (nr/nt) database. Phylogenetic trees based on 16S rRNA gene (Figure S1) were constructed by Mega-X using the maximum likelihood method with the Tamura-Nei model. The 16S sequence of *Akkermansia muciniphila* (accession number: NR_074436.1; the type species of the genus *Akkermansia*, a mesophilic mucin degrading Verrucomicrobiota representative from the human gut) was used as an outgroup for comparison. The genetic information of mesophilic Verrucomicrobiota (*Verrucomicrobium spinosum* DSM 4136 (NR_026266.1)), mesophilic verrucomicrobiotal methanotrophs (*Verrucomicrobium* sp. LP2A (AM900834.1), and 3C (NR_126313.2)), and acidophilic verrucomicrobiotal methanotrophs (*M. infernorum* V4 (EU223931.1), *M. fumariolicum* SolV (EF591088.1), *M. kamchatkense* Kam1 (EF127896.1), and Ca. *Methylacidiphilum* sp. Yel (LXQB01000011.1)) were also used to generate the phylogenetic tree.

### 2.3. DNA Extraction, gDNA Library Preparation, and Genome Sequencing

The next step was to purify pure cultures of the Ca. *Methylacidiphilum* isolates from the Yellowstone enrichments; however, during resuscitation from glycerol stocks, the Ca. *Methylacidiphilum* strain was overgrown by co-cultured *Alicyclobacillus acidocaldarius* that grew on the residual glycerol transferred with the stock. This occurred a number of times while we determined the cause and depleted our stocks. By relaxing growth conditions to pH 3.5 and temperature 55 °C, we inadvertently made the overgrowth problem worse. For this reason, enrichment cultures showing the high homogeneity with Ca. *Methylacidiphilum* were used for the DNA extraction.

The enriched methanotrophic cultures (NL01A and NG05G) were used for the extraction of genomic DNA (gDNA) using a DNeasy^®^ Blood & Tissue Kit (Qiagen, Valencia, CA, USA) according to the manufacturer’s instructions. DNA concentrations were then measured by Qubit with a dsDNA HS assay kit (Life Technologies, Thermo Fisher Scientific Inc., CA, USA). DNA concentrations of NL01A from Nymph Lake and NG05G from Norris Geyser Basin were 3.28 µg/mL (total 65.6 ng) and <0.5 µg/mL (less than 15 ng), respectively. Sample NG05G with a low DNA amount was treated with the Ovation UltraLow V2 DNA-seq Library preparation kit (Tecan Genomics, Redwood City, CA, USA), which is used to rescue samples with picogram amounts of DNA. The shotgun gDNA library for the NL01A sample was prepared with a Hyper library construction kit (Kapa Biosystem, MA, USA). gDNA libraries were quantitated by qPCR and confirmed by gel electrophoresis. Sequencing was carried out with 251 cycles from each end of the fragments on a NovaSeq 6000 (Illumina, Inc., San Diego, CA, USA) using a NovaSeq SP reagent kit (Illumina). Fastq files were generated and demultiplexed with the bcl2fastq v2.20 conversion software (Illumina). A total of 73,297,671 paired-end reads with read size of 250 × 2 nt were obtained (Step 3 in Figure 1A). The Phred quality-scores line in fastq files used an ASCII offset of 33 known as Sanger scores.

### 2.4. Computational Pipeline for Finding Verrucomicrobiotal Genome Sequence

To determine the best verrucomicrobiotal genome sequence bin, we used the pipeline presented in Figure 1B. Processed paired-end genome sequencing reads were subject to de novo metagenome assembly using MEGAHIT ver. 1.2.9 (https://github.com/voutcn/megahit, accessed on 27 April 2021) and MetaSpades ver. 3.14.1 (https://github.com/ablab/spades, accessed on 27 April 2021). Contigs shorter than 1 kb were dropped from the pool. Original reads were mapped to the contigs using Bowtie2 ver. 2.4.2 (https://github.com/BenLangmead/bowtie2, accessed on 27 April 2021) and BWA ver. 0.7.17 (https://github.com/lh3/bwa, accessed on 27 April 2021), and the read coverage of each contig was calculated. Contigs were identified based on the presence of repeated sequences on both ends using the protocol previously described in Jorgensen et al. (2014) [30]. Automated binners MetaBAT2 ver. 2.12.1 (https://guix.gnu.org/en/packages/metabat-2.12.1/, accessed on 27 April 2021), MaxBiH ver. 2.2.7 (https://sourceforge.net/projects/maxbin2/files/, accessed on 27 April 2021) and CONCOCT ver. 1.1.0 (https://github.com/BinPro/CONCOCT, accessed on 27 April 2021) were executed using default parameters. All bins were further refined and reassembled through the MetaWrap pipeline [31]. Final bins were submitted to the CheckM ver. 1.0.7 (https://github.com/Ecogenomics/CheckM/wiki, accessed on 27 April 2021) for the quality check, and then we found verrucomicrobiotal genomes based on the presence of marker gene *pmoA* (Step 4 in Figure 1A). From the 18 bins predicted to belong to the phylum Verrucomicrobiota (Table S1), five bins were selected as best bin candidates based on completeness, contamination rate, genome size, GC contents, and open reading frames (Table S2).

### 2.5. Comparative Genome Analysis

The selected genome bins were mapped to *M. fumariolicum* SolV, *M. kamchatkense* Kam1, and *M. infernorum* V4 reference genomes using BWA. The ANI was calculated by comparison of the sequences with other methanotroph strains (gammaproteobacterial methanotrophs: *Methylomicrobium buryatense* 5G, *Methylococcus capsulatus* Bath, and *Methylomonas methanica* MC09; alphaproteobacterial methanotrophs: *Methylosinus trichosporium* OB3B; verrucomicrobiotal methanotrophs: *M. fumariolicum* SolV, *M. kamchatkense* Kam1, *M. infernorum* V4, *M. fumariolicum* Fur, *M. fumariolicum* Ice, *M. fumariolicum* Rib, *M. fumariolicum* Fdl, Ca. *Methylacidiphilum* sp. Yel, and Ca. *Methylacidiphilum* sp. Phi). All available genome sequences in the RefSeq database (NCBI) were adopted as reference genomes, and genomic data were obtained from the NCBI site. Python ver. 3.7 with Seaborn library was used for the visualization of the ANI values. In addition, a phylogenetic tree based on the functional gene (*pmoA*, Figure S2; *pmoC*, Figure S3) was generated by the NCBI blastp using a maximum likelihood method with the NCBI non-redundant protein sequences (nr) database.

### 2.6. Metabolic Pathways and Relative Gene Alignments Analysis

Open reading frames on the assembled contigs were identified and translated into amino acid sequences using PROKKA (ver. 1.14.6, accessed on 27 April 2021). Clusters of orthologous group (COG) functional categories of the annotated genes were characterized by eggNOG-mapper (ver. 2.0) analysis. In order to analyze metabolic pathways more specifically, genomes of the four reference strains *M. fumariolicum* SolV, *M. infernorum* V4, *M. kamchatkense* Kam1, and Ca. *Methylacidiphilum* sp. Yel were compared with those of Ca. *Methylacidiphilum* sp. YNP IV newly assembled in this study. We searched for the presence or absence of functional genes predicting metabolic pathways associated with methane oxidation (KEGG map number 00680), the Calvin–Benson–Bassham (CBB) pathway for CO_2_ fixation (00710), and nitrogen metabolism (00910). NCBI BLAST was used to highlight protein sequence identities among the strains with default settings. Local genome and alignments were analyzed and confirmed with Geneious Prime 2021. Identities of the *pmoC*s in the Ca. *Methylacidiphilum* sp. YNP IV and its phylogeny tree were developed by MUSCLE multiple sequence alignment. Hydrogenases were classified with HydDB [32], and the related gene set was identified manually. Antimicrobial resistance genes were identified based on the comprehensive antibiotic resistance database (CARD; https://card.mcmaster.ca, accessed on 27 April 2021). Defense mechanisms including acid and heat stress responses, heavy metal resistance, as well as CRISPR system-associated genes and inosine monophosphate dehydrogenase (IMDPH)-encoding genes were also identified in the annotated genome file.

### 2.7. Nucleotide Sequence Accession Numbers and Data Availability

The assembled genome has been deposited at the NCBI under the submission number SUB9785567 (BioProject: PRJNA737000; BioSample: SAMN19677571). The version described in this manuscript is the first version.

## 3. Results and Discussion

### 3.1. Identification of Verrucomicrobiotal Methanotrophs from Yellowstone Hot Spring Samples

Six samples were collected from Nymph Lake (NL01A, NL03, NL10, and NL13), Norris Geyser Basin (NG05G), and Mud Volcano (MV04) in Yellowstone National Park (Step 1 in Figure 1A). After several transfers to enrich for the methanotroph at pH 2.0 and 60 °C with the addition of REE trace element supplement (0.2 µM of lanthanum and cerium), we obtained cultures from NL01A and NG05G (Step 2 in Figure 1A) that yielded 16S rRNA gene sequences with more than 98% identity to *M. fumariolicum* SolV (Figure S1).

### 3.2. Metagenomic Approaches to Identify Verrucomicrobiotal Genomes

**Figure 2:**
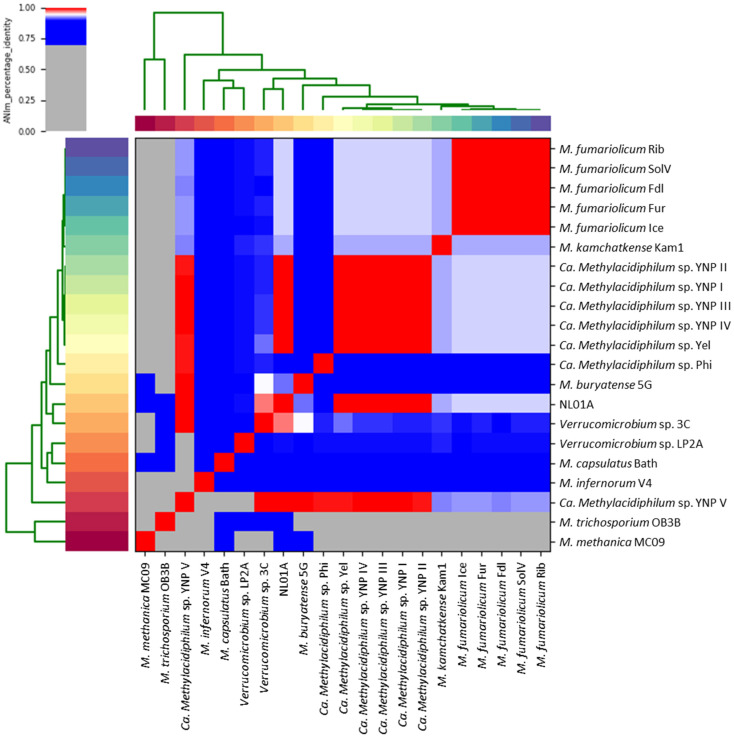
Heatmap generated from the comparative analysis of the average nucleotide identity (ANI) between the best bins produced from the sample NL01A and other methanotroph strains. Pyani version 0.2.10 was used for the visualization. Gamma-proteobacterial methanotrophs: *M. buryatense* 5G, *M. capsulatus* Bath, *M. methanica* MC09; alpha-proteobacterial methanotrophs: *M. trichosporium* OB3B; Verrucomicrobiotal methanotrophs: *M. fumariolicum* SolV, *M. kamchatkense* Kam1, *M. infernorum* V4, *M. fumariolicum* Fur, *M. fumariolicum* Ice, *M. fumariolicum* Rib, *M. fumariolicum* Fdl, Ca. *Methylacidiphilum* sp. Yel, and Ca. *Methylacidiphilum* sp. Phi.

DNA extraction from the enrichment necessitated an alternative approach that entailed obtaining the whole genome sequence of Verrucomicrobiota using a metagenomic approach in which the target organism was a minority member in the enriched consortium (Step 3 in Figure 1A). Through the process described in Figure 1, the putative Ca. *Methylacidiphilum* genomes were found in sample NL01A but were not found in sample NG05G (Step 4 in Figure 1A). As the extracted DNA from the Yellowstone Hot Spring sample enrichments were derived from a microbial consortium, genome bins were generated through the combination of different programs as described in Figure 1B. The comparative analysis of the ANI between the produced bins from the samples and reference strains of Ca. *Methylacidiphilum* sp., including *M. buryatense* 5G, *M. capsulatus* Bath, *M. methanica* MC09, *M. trichosporium* OB3B, *M. fumariolicum* SolV, *M. kamchatkense* Kam1, *M. infernorum* V4, *M. fumariolicum* Fur, *M. fumariolicum* Ice, *M. fumariolicum* Rib, *M. fumariolicum* Fdl, Ca. *Methylacidiphilum* sp. Yel, and Ca. *Methylacidiphilum* sp. Phi (Figure 2) showed a high possibility of the presence of a Ca. *Methylacidiphilum* genome.

In order to extract accurate target sequences, two assembly tools, two alignment tools, and three binning tools were used. Bins from the sample NL01A, which contained the putative Ca. *Methylacidiphilum* genome, were refined and reassembled with the MetaWRAP pipeline [25]. A total of 18 different combinations were carried out, and verrucomicrobiotal genomes were identified with the functional marker gene *pmoA* (Table S1), which encodes the highly conserved alpha subunit of pMMO and is often used as a biomarker for identifying methanotrophs [33,34]. The completeness of the 18 bins ranged from 90.70 to 98.65%, and the contamination rate ranged from 1.69 to 3.45%. Among the produced bins, the best bin candidates (YNP I to V) were selected based on the completeness of the genome (more than 97%), contamination rate, genome size, GC contents, and open reading frames (ORFs) (Tables S1 and S2).

### 3.3. Selection of the Best Bin as a Verrucomicrobiotal Genome

For the purpose of selecting the best bin from the candidates derived from the sample NL01A, comparative genomics using the complete genome of *M. fumariolicum* SolV, *M. infernorum* V4, and *M. kamchatkense* Kam1 was performed. When the sequencing reads of each bin were mapped to the reference genomes, the overall alignment rate was as follows: *M. fumariolicum* SolV (63.82 to 91.19%), *M. infernorum* V4 (10.44 to 15.03%), and *M. kamchatkense* Kam1 (62.63 to 89.04%). The highest alignment rate was shown with *M. fumariolicum* SolV, which is consistent with our 16S rRNA gene identity analysis. Among the bins, sequences in YNP IV showed the highest mapping reads to *M. fumariolicum* SolV (40,821,695). For comparison, ANI values were also calculated not only with the previously isolated verrucomicrobiotal methanotrophs (*M. fumariolicum* SolV, *M. infernorum* V4 and *M. kamchatkense* Kam1) but also gamma-proteobacterial (*M. buryatense* 5G, *M. capsulatus* Bath and *M. methanica* MC09), alpha-proteobacterial (*M. trichosporium* OB3B), and the recently identified verrucomicrobiotal methanotrophs (*M. fumariolicum* Ice, *M. fumariolicum* Fur, *M. fumariolicum* Rib, *M. fumariolicum* Fdl, Ca. *Methylacidiphilum* sp. Phi, and Ca. *Methylacidiphilum* sp.Yel). The bins showed around ANI values of 0.80 for the type I, II, and *M. infernorum* genomes except for *M. methanica* MC09 and *M. trichosporium* OB3B (ANI value of 0). Otherwise, they showed ANI values of 0.93 to 0.94 for *M. fumariolicum* SolV. The highest number was shown with Ca. *Methylacidiphilum* sp. Yel with an ANI value of 0.998 between Ca. *Methylacidiphilum* sp. Yel and the bin YNP IV (Figure 2).

By considering the general features of the genome and comparative genome analysis, the best bin of Verrucomicrobiota was determined as YNP IV. We named it Ca. *Methylacidiphilum* sp. YNP IV, which had the following genomic features: total sequence length (2.47 Mbp), number of contigs (82 contigs), GC contents (41.26%), ORFs (2392), and number of the pmo genes (three *pmoA*s, four *pmoB*s, and five *pmoC*s). A comparison of the bins and nine reference strains is shown in Table 1. The total sequence length, GC content, number of coding sequence (CDS), rRNA, and tRNA were similar among the strains. The robust phylogeny tree of the *pmoA* in the selected genomes (Figure S2) also revealed a close relationship between Ca. *Methylacidiphilum* sp. YNP IV and the *pmoA* gene in Ca. *Methylacidiphilum* sp. Yel as shown in the ANI results in Figure 2.

### 3.4. Clusters of Orthologous Groups (COGs)

**Figure 3:**
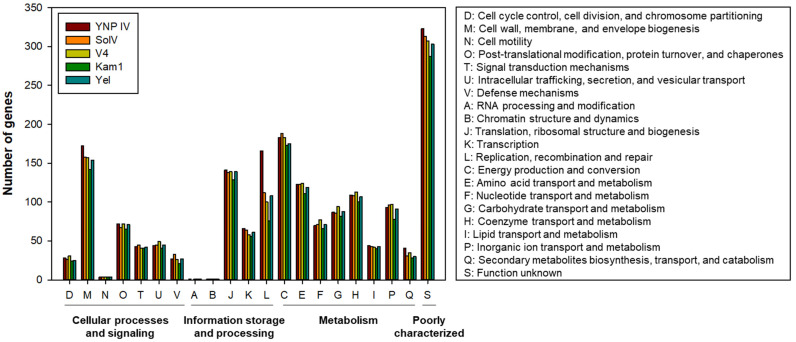
Cluster of orthologous group (COG) functional category of the annotated genes in Ca. *Methylacidiphilum* sp. YNP IV, *M. fumariolicum* SolV, *M. infernorum* V4, *M. kamchatkense* Kam1, and Ca. *Methylacidiphilum* sp. Yel. COG categories were classified by eggNOG-mapper ver. 2.0 (accessed on 27 April 2021).

COG categories of the annotated genes (1838 genes in the Ca. *Methylacidiphilum* sp. YNP IV, 1752 genes in *M. fumariolicum* SolV, 1887 genes in *M. infernorum* V4, 1704 genes in *M. kamchatkense* Kam1, and 1705 genes in Ca. *Methylacidiphilum* sp. Yel) are shown in Figure 3. Specifically, it was determined that the Ca. *Methylacidiphilum* sp. YNP IV genome includes more genes belonging to the COG category of M (cell wall, membrane, and envelope biogenesis), L (replication, recombination, and repair), and Q (secondary metabolites biosynthesis, transport, and catabolism) as compared to the other reference genomes.

### 3.5. Methanotrophic and Central Metabolic Pathways

**Figure 4:**
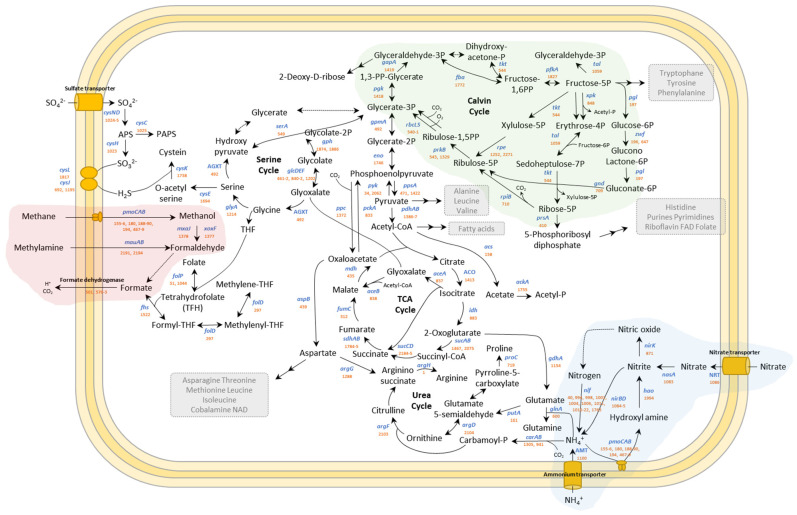
Reconstruction of methanotrophic and central metabolism pathways of Ca. *Methylacidiphilum* sp. YNP IV. For each predicted reaction, blue letters indicate the generic gene name, and orange numbers indicate gene identifier (“PPMHHGHK” prefix is omitted). The methane oxidation pathways, CO_2_ fixation through the CBB pathway, and nitrogen metabolism are shown in red, green, and blue, respectively.

The Ca. *Methylacidiphilum* sp. YNP IV contigs showed most of the key genes for the central metabolism pathways including methane oxidation (red), CBB pathways for CO_2_ fixation (green), and nitrogen metabolism (blue) (Figure 4).

#### 3.5.1. Pathways Associated with Methane Metabolism

**Figure 5:**
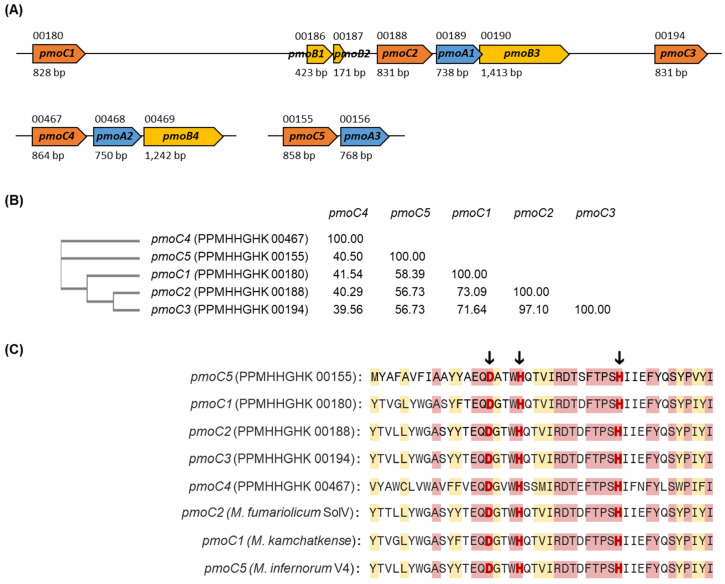
(**A**) Alignment of the *pmo* cluster in Ca. *Methylacidiphilum* sp. YNP IV. The numbers above and under the arrow are locus tag numbers without prefix (PPMHHGHK) and gene size. (**B**) Phylogenetic tree and percent identity of *pmoC*s in the YNP IV. (**C**) Alignment of the *pmoC*s from Ca. *Methylacidiphilum* sp. YNP IV and *M. fumariolicum* SolV, *M. kamchatkense* Kam1, and *M. infernorum* V4. *pmoC* numbers are given followed by locus tag without prefix. The conserved copper-binding motif (DxxxH(x_12_)H) is indicated by black arrows and red letters.

In the methane metabolism pathway, we identified three *pmo* clusters in the Ca. *Methylacidiphilum* sp. YNP IV genome, which was also reported previously for *M. kamchatkense* Kam1. As a comparison, *M. fumariolicum* SolV and *M. infernorum* V4 had two *pmo* clusters [19]. Ca. *Methylacidiphilum* sp. YNP IV encodes a unique *pmoC*A cluster like *M. kamchatkense* Kam1, which is not present in *M. fumariolicum* SolV and *M. infernorum* V4 (Figure 5). In brief, Ca. *Methylacidiphilum* sp. YNP IV genome contains two *pmoC*AB operons, along with a truncated *pmoC*A operon and two separate *pmoC* genes in separate loci (PPMHHGHK 00180 and 00194). Awale et al. (2021) showed that the *pmoCAB3* operon is involved in acetone metabolism [18]. However, in the present study, *pmoCAB3* was absent or it may be represented by the truncated *pmoCA* or other two separate *pmoC* genes. All *pmo* genes in the clusters showed 41.60 to 100% of identity against the *pmo* genes in *M. fumariolicum* SolV, *M. infernorum* V4, *M. kamchatkense* Kam1, and Ca. *Methylacidiphilum* sp. Yel (Table S3).

In our genome assembly, the presence of multiple copies of the *pmoC* genes in Ca. *Methylacidiphilum* sp. YNP IV was observed, which may be the result of gene duplication. The *pmoC* genes, located in close proximity, showed a higher percent identity to each other as described before. *PmoC* binds to copper and activates the pMMO enzyme. The copper-binding motif (DxxxH(x12)H) was conserved in all *pmoC*s, as well as the three reference strains (Figure 5).

A homolog of *xoxF* (PPMHHGHK 01377), encoding the lanthanide-dependent methanol dehydrogenase XoxF, and *xoxJ* (PPMHHGHK 01378), encoding the methanol oxidation protein XoxJ, were also identified in Ca. *Methylacidiphilum* sp. YNP IV genome. The product of the fused *xoxGJ* genes was purified in *M. fumariolicum* SolV [35]. Likewise, the *xoxG* gene (PPMHHGHK 01379) was present downstream of *xoxJ* in YNP IV. The genes encoding for enzymes of PQQ biosynthesis, which is the cofactor for methanol dehydrogenase, are found in the genome assembly (Pqq BCDE cluster, PPMHHGHK 00565-8), while the *pqqA* gene, encoding coenzyme PQQ synthesis protein A, was not detected. Although formaldehyde dehydrogenase was missing in the Ca. *Methylacidiphilum* sp. YNP IV genome, it seems that the Xox-type MDH encoded by *xox* contributes to the oxidation of formaldehyde in verrucomicrobiotal methanotrophs [36]. Ca. *Methylacidiphilum* sp. YNP IV has the gene encoding an alternative dihydropteroate synthase (*folp*) [37], while it does not encode dihydrofolate reductase (FolA), which is involved in the last step of folate synthesis that is important for DNA synthesis.

**Figure 6:**
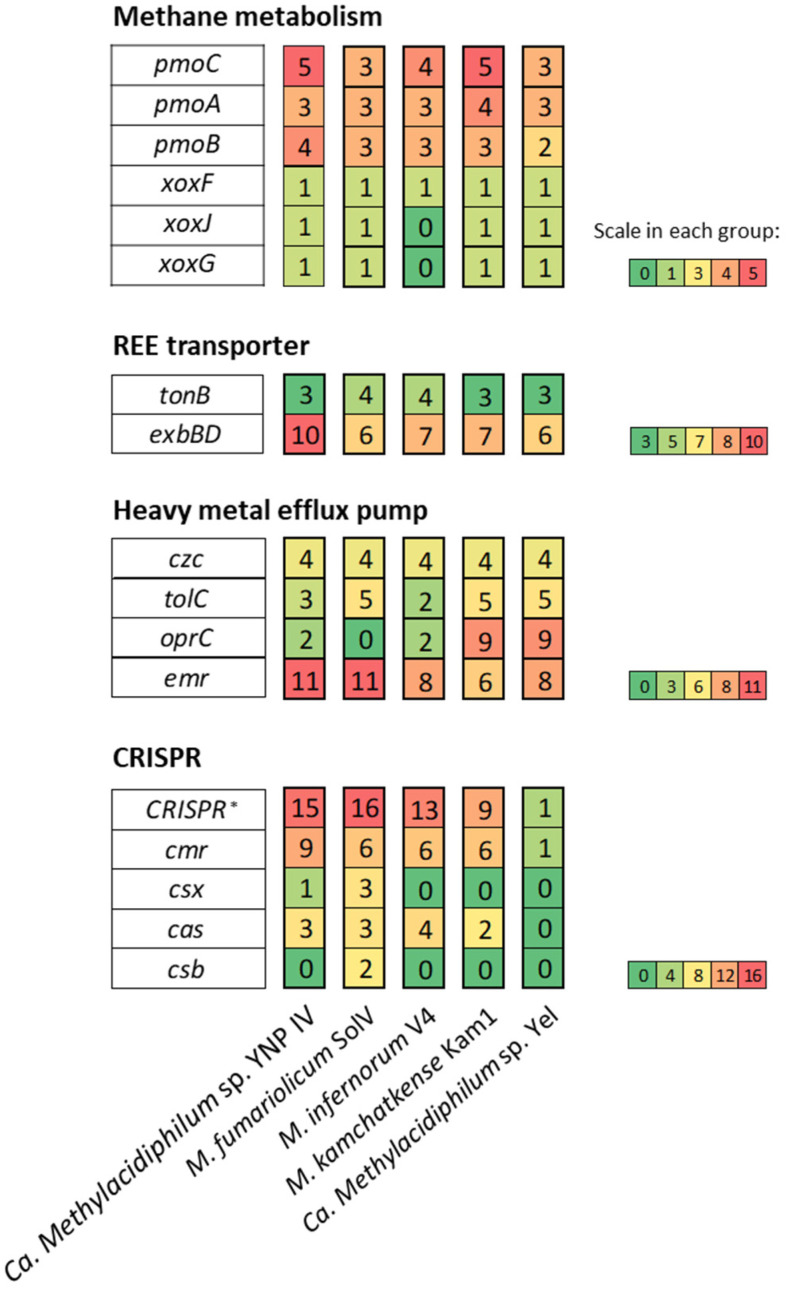
Comparison of the genes associated with methane metabolism, REE transporter, heavy metal efflux pump, and CRISPR in Ca. *Methylacidiphilum* sp. YNP IV, *M. fumariolicum* SolV, *M. infernorum* V4, *M. kamchatkense* Kam1, and Ca. *Methylacidiphilum* sp. Yel. Scale representing the number of gene sequences is presented for each functional group as green (low) to red (high). * CRISPR-associated protein-encoding genes including *cmr*, *cas*, *csx*, and *csb*.

REEs such as lanthanide and cerium are essential for methanotrophic life in volcanic environments [23]. TonB-dependent transporters are outer membrane importers relying on the proton motive force created by the TonB-ExbB-ExbD complex [38]. In this study, three *tonB* genes and ten *exbBD* genes were detected in the genome of Ca. *Methylacidiphilum* sp. YNP IV (Figure 6).

#### 3.5.2. Pathways Associated with Carbon Metabolism (CO_2_ Fixation)

Verrucomicrobiotal methanotrophs use the CBB pathway to assimilate carbon from methane oxidation [37,39,40], while other methanotrophs assimilate formaldehyde through the ribulose monophosphate (RuMP) pathway or serine pathway [41]. The Ca. *Methylacidiphilum* sp. YNP IV genome assembly encodes a full CBB pathway for CO_2_ fixation [11,13,37], which means it would be able to obtain carbon by fixing CO_2_ rather than using formaldehyde [39]. We found large numbers of genes associated with the CBB pathway; however, there were notable differences between Ca. *Methylacidiphilum* sp. YNP IV and the other methanotrophs. We found that Ca. *Methylacidiphilum* sp. YNP IV genome did not have the *tpi* gene encoding triose phosphate isomerase for conversion of glyceraldehyde-3P to dihydroxyacetone phosphate. Instead, it appears to convert glyceraldehyde-3P to fructose-1,6-PP using fructose-bisphosphate aldolase (PPMHHGHK 01772). It also possesses the *prkB* gene encoding phosphoribulokinase, the essential enzyme of the CBB cycle, which did not show sequence matches in *M. fumariolicum* SolV and *M. kamchatkense* Kam1.

#### 3.5.3. Pathways Associated with Nitrogen Metabolism

Apart from methane metabolism, nitrogen fixation is widely found among methanotrophs and is identified by the presence of *nifH* genes [42]. *M. fumariolicum* SolV and *M. infernorum* V4 have a complete gene set for nitrogen fixation [30,37]. In this study, the genome information revealed Ca. *Methylacidiphilum* sp. YNP IV appears to be able to use nitrogen (N_2_), nitrate, and ammonium as nitrogen sources by containing the *nifHDKENB* cluster as well as in *M. fumariolicum* SolV, *M. infernorum* V4, *M. kamchatkense* Kam1, and Ca. *Methylacidiphilum* sp. Yel [19]. Ca. *Methylacidiphilum* sp. YNP IV also encodes nitrogen fixation protein NifT and NifZ and the ferredoxin-like protein, which are not present in *M. fumariolicum* SolV.

Intracellular ammonium produced by nitrogen fixation or transported by the ammonium transporter (PPMHHGHK 01100) can be oxidized by the pMMO enzyme, resulting in the formation of the intermediate hydroxylamine [5,43,44]. While ammonia-oxidizers are able to deliver electrons to the quinone pool to generate energy from hydroxylamine oxidation [45], methanotrophs are unable to produce energy from this metabolic pathway [46]. Instead, methanotrophs rapidly remove the toxic hydroxylamine through detoxification achieved by conversion of hydroxylamine to nitrite using a hydroxylamine dehydrogenase *hao* (PPMHHGHK 01994). Nitrate, which can also be imported by the nitrate transporter (*NRT*, PPMHHGHK 01086) in the membrane, is converted to nitrite by nitrate reductase (*nasA*, PPMHHGHK 01083). *nirK* (PPMHHGHK 00871), *nirBD* (PPMHHGHK 01084-5), and *norCB* (PPMHHGHK 01443-4) in YNP IV are essential for the conversion of nitrite to nitric oxide or ammonium, which is the same as *M. fumariolicum* SolV.

### 3.6. Hydrogenases

Methanotrophs are believed to conserve reducing equivalents and produce energy by using the hydrogen produced during methane oxidation and nitrogen fixation [3]. This hydrogen turnover is catalyzed by hydrogenases. It has been reported that the hup-type hydrogenases of the verrucomicrobiotal methanotrophs belong to the membrane-bound H_2_-uptake NiFe-hydrogenases, and the hhy-type hydrogenase belongs to group 1 h/5 hydrogenases [2]. The *hupB* gene (PPMHHGHK 02151), which encodes for the large subunits of group 1d NiFe-hydrogenase, was also found in the Ca. *Methylacidiphilum* sp. YNP IV genome (Figure S4), while *M. infernorum* RTK17.1 and *M. fumariolicum* SolV contain genes for group 1d and 3b [27,47]. The products of six genes, *hypABCDEF*, are necessary for the maturation and incorporation of metal cofactors in the active site of NiFe hydrogenases [48]. A full *hypABCDEF* gene set was found in Ca. *Methylacidiphilum* sp. YNP IV (PPMHHGHK 00336-9, 00341, and 01479).

### 3.7. Defense Mechanisms

#### 3.7.1. Acid and Heat Stress Responses

Verrucomicrobiotal methanotrophs are extreme thermoacidophiles as they colonize volcanic and geothermal environments. Acid and heat stress-related genes are likely critical for survival and growth of these bacteria. In the present study, we identified proton sequestration and repair of protein/DNA-related genes in Ca. *Methylacidiphilum* sp. YNP IV (Figure S5). It has genes that mediate glutamate decarboxylation (PPMHHGHK 01732) and arginine decarboxylation for the removal of protons from the cytosol (PPMHHGHK 01816). It is well known that the arginine-dependent acid resistance system consists of arginine decarboxylase encoded by *adiA* and an arginine/agmatine antiporter encoded by *adiC* [49]. However, the genome of Ca. *Methylacidiphilum* sp. YNP IV does not include *adiA*. Instead, it has a biosynthetic *speA* that catalyzes the biosynthesis of agmatine from arginine, which is consistent with a previous study on *M. fumariolicum* SolV, *M. infernorum* V4, and *M. kamchatkense* Kam1 [19]. The Ca. *Methylacidiphilum* sp. YNP IV encodes heat shock protein HtpX, ATP-dependent Clp protease, molecular chaperone GrpE, DnaJ, and DnaK, and chaperonin GroEL and GroES involved in protein protection and repair. It was shown to possess the full set of *uvrABCD* and *recA*, which provide a mechanism to repair DNA damage. The UvrABCD system mediates the bacterial nucleotide excision repair system, which plays a role in removing a large array of DNA lesions [50]. RecA is required for the bypass of mutagenic DNA lesions by the stress response. These genes likely contribute to the survival of the Ca. *Methylacidiphilum* sp. YNP IV under the extreme conditions in which it lives.

#### 3.7.2. Heavy Metal and Antimicrobial Resistance

Due to the increased solubility of heavy metals under acidic pH conditions [51,52,53], acidophilic methanotrophs have to cope not only with low pH but also with heavy metal toxicity. It was reported that methanotrophs isolated from acidic forest soils encode elaborate systems for heavy metal efflux including resistance–nodulation–cell division (RND) transporters and major facilitator superfamily (MFS) transporters [52]. In addition, genes for copper resistance (*copA*, PPMHHGHK 00226) for metal homeostasis [4] and arsenate reductase (*arsC*, PPMHHGHK 00,491 and 01323) linked to the heavy metal efflux pump [4] were also identified in Ca. *Methylacidiphilum* sp. YNP IV (Supplementary Excel 2). Among the antimicrobial resistance mechanism-related genes in Ca. *Methylacidiphilum* sp. YNP IV (Figure S6), those associated with antibiotic efflux were the highest (57.7%), followed by antibiotic target alteration (22.6%), in terms of resistance mechanism. The top three antimicrobial resistance gene families by number were RND antibiotic efflux pumps, ABC antibiotic efflux pumps, and MFS antibiotic efflux pumps (Table S4), which are frequently observed in other acidophilic microorganisms [52,54,55]. Specifically, four *czc* (heavy metal efflux pump, CzcA family), three *tolC* (outer membrane protein TolC), two *oprC*, (copper transport outer membrane porin OprC), and eleven *emr* genes (RND transporter) were identified (Figure 6).

#### 3.7.3. CRISPR

CRISPR systems are involved in antiviral defense mechanisms [56,57]. Viral predation is an important ecological pressure for the survival of bacteria in acidic conditions. Two different CRISPR-Cas systems of acidophilic methanotrophs were reported which may increase bacterial resistance to the viral infection [52]. It was also found that there is a type III CRISPR-Cas adaptive immune system in *M. fumariolicum* SolV, *M. infernorum* V4, and *M. kamchatkense* Kam1 [19]. However, no CRISPR loci were reported in the genome of Ca. *Methylacidiphilum* sp. Yel [16]. We detected two CRISPR loci (Table 1) with nine *cmr* genes encoding Cas module repeat-associated mysterious proteins (Cmr) and one *csx3* gene, as well as three *cas* genes (Figure 6, Supplementary Excel 2). This finding supports the previous report that verrucomicrobiotal methanotrophs have type III-B CRISPR-Cas modules.

#### 3.7.4. IS607-Family Transposase and MerR Family DNA-Binding Transcriptional Regulator

Interestingly, after initial annotation with EggNog mapper, 24 copies of the putative *guaB* gene, which is essential for the metabolism of purine nucleotides [58,59], were recovered in the Ca. *Methylacidiphilum* sp. YNP IV genome assembly. This was resolved using a manual recheck of each putative gene with NCBI database that confirmed only one *guaB* gene in the assembly. Instead, the other putative *guaB* genes were IS607-family transposases (n = 14) and MerR family DNA-binding transcriptional regulator (n = 9)-related genes, which are unusual serine site-specific recombinase and the major regulators that respond to environmental stimuli such as heavy metals or antibiotics, respectively.

## 4. Summary and Conclusions

In this study, we present the sequencing, assembly, and reconstruction of the genome of Ca. *Methylacidiphilum* sp. YNP IV isolated from Nymph Lake in Yellowstone National Park in the USA. This was achieved by metagenomic sequencing of a mesophilic enrichment at 60 °C and pH 2.0 of a sediment sample obtained from Nymph Lake. The genomic features of Ca. *Methylacidiphilum* sp. YNP IV show three *pmo* clusters associated with the methane metabolism as well as the conserved copper-binding motif in all *pmoC*s, which is essential for the pMMO activation. Ca. *Methylacidiphilum* sp. YNP IV encodes a full gene set for CO_2_ and N_2_ fixation used to synthesize the biomass under autotrophic growth conditions. The presence of *hup* and *hypABCDEF* genes suggests the group 1d NiFe-hydrogenase plays a role in providing energy for methanotrophic growth. The *tonB-exbBD* gene set was found in Ca. *Methylacidiphilum* sp. YNP IV, which is required for the REE lanthanide transportation in order to activate the XoxF-type methanol dehydrogenase. Various genes related to proton sequestration, repair of protein or DNA damage, heavy metal efflux system and resistance, and CRISPR systems were also found in Ca. *Methylacidiphilum* sp. YNP IV. This contributes to the growth and survival of the methanotrophs under environmental conditions of high temperature and low pH.

By definition, a metagenome-assembled genome (MAG) is a single-taxon assembly based on one or more binned metagenomes that has been asserted to be a close representation to an actual individual genome (that could match an already existing isolate or represent a novel isolate). MAGs are typically incomplete and may contain contigs from multiple strains or species due to challenges in distinguishing between related community members both in the binning and assembly processes [60]. This is one of the limitations of the MAG approach, which is entirely dependent on sequencing and bioinformatics to assemble what is likely a composite or mosaic genome from several different strains. Therefore, we are unable to state that these bins represent a single species or strain. Nevertheless, the MAG process works best when applied to extreme environments such as the hot springs and volcanic mud pots sampled in this study, which have limited bacterial diversity. In the future, we intend to overcome this limitation to our work by obtaining fresh samples from Yellowstone National Park to repeat the isolation work in order to obtain pure cultures of the *Methylacidiphilum* species for genomic sequencing and analysis, as well as future functional work.

Further biochemical and genomic studies using cultivated strains of *Methylacidiphilum* sp. are expected to provide insight into the adaptation of methane oxidation pathways to acidic (pH 1–2) and thermophilic (>60 °C) environmental conditions. Proteins encoded in these sequenced genomes are expected to provide biotechnological insights, advances, and catalysts for the biological conversion of gas to liquids utilizing methane as feedstock. In conclusion, the discovery of the new genome assembly of a thermoacidophilic methanotrophic Verrucomicrobiota from a geothermal environment expands the diversity of biological methane conversion and explains its adaptation and survival in extreme environments.

## Figures and Tables

**Table 1:** Comparison of the genomic properties of Ca. *Methylacidiphilum* strains.

Reference	Total Sequence Length	GC Content	Number of Contigs	Contig N50	CDS	rRNA	tRNA	CRISPR
*Ca. Methylacidiphilum* sp. YNP IV	2,467,065 bp	41.3%	82	76,993	2288	3	49	2
*M. fumariolicum* Ice	2,376,773 bp	41.0%	91	56,162	2128	3	48	2
*M. fumariolicum* Fur	2,391,355 bp	41.0%	101	53,961	2145	3	48	2
*M. fumariolicum* Rib	2,392,263 bp	41.0%	113	53,912	2152	3	48	2
*M. fumariolicum* Fdl	2,381,209 bp	41.0%	96	53,912	2136	3	48	2
*Ca. Methylacidiphilum* sp. Phi	2,337,855 bp	41.4%	231	64,983	2112	3	50	3
*Ca. Methylacidiphilum* sp. Yel	2,250,350 bp	41.1%	107	46,532	2073	3	47	-
*M. fumariolicum* SolV (NZ_LM997411.1)	2,476,671 bp	40.9%	1	-	2296	3	48	2
*M. kamchatkense* Kam1 (NZ_CP037899.1)	2,202,032 bp	40.4%	1	-	1962	3	46	1
*M. infernorum* V4 (NC_010794.1)	2,287,145 bp	45.5%	1	-	2055	3	46	3

## Data Availability

Not applicable.

## Supplementary Materials

The following supporting information can be downloaded at https://www.mdpi.com/article/10.3390/microorganisms10010142/s1, Table S1. Bins from the metagenome data of the sample NL01A and identification of Verrucomicrobiota; Table S2. Best bin candidates for identifying Verrucomicrobiota from the sample NL01A after binning, bin refinement, and re-assembly; Table S3. Genes associated with methane oxidation, CO_2_ fixation, and nitrogen metabolism in strains YNP IV, SolV, V4, Kam1 and Yel; Table S4. Number of antimicrobial resistant (AMR) gene families in the YNP IV genome assembly; Figure S1. Phylogenetic analysis of Yellowstone hot spring samples and Verrucomicrobiota strains by the Maximum Likelihood tree using the Tamura-Nei model of the 16S rRNA gene sequences. Accession numbers for each reference bacteria are included in parentheses in the figure; Figure S2. Phylogenetic analysis of the *pmoA* gene in YNP IV strain obtained using the Maximum Likelihood method. The tree is constructed from *pmoA* gene sequences of other references that have been obtained from the NCBI database. The percentage of trees where the associated taxa clustered together is presented next to the branches. Evolutionary analyses were conducted in MEGA-X; Figure S3. Phylogenetic analysis of the *pmoC* gene in YNP IV strain obtained using the Maximum Likelihood method. The tree is constructed from *pmoA* gene sequences of other references that have been obtained from the NCBI database. The percentage of trees where the associated taxa clustered together is presented next to the branches. Evolutionary analyses were conducted in MEGA-X; Figure S4. Hydrogenase genes related to methane and nitrogen metabolism and transportation of rare earth elements in the YNP IV. The numbers above and under the arrow are locus tag numbers without prefix (PPMHHGHK) and gene size; Figure S5. Acid and heat stress-associated genes in the YNP IV. (A) Proton sequestration. (B) Protection and repair of protein and DNA. Blue letters represent gene names and orange numbers indicate locus tag numbers without prefix (PPMHHGHK); Figure S6. Pie graph showing the percentage of antimicrobial resistance mechanism related genes in the YNP IV genome assembly.
