# Supplementary material for: Genome Sequence of a Thermoacidophilic Methanotroph Belonging to the Verrucomicrobiota Phylum from Geothermal Hot Springs in Yellowstone National Park: A Metagenomic Assembly and Reconstruction
Source: Microorganisms. 2022 Jan 11;10(1):142. doi: 10.3390/microorganisms10010142 (PMC8779874; doi:10.3390/microorganisms10010142)
Supplement: Supplementary file 1 [file microorganisms-10-00142-s001.zip › microorganisms-1471770-supplementary.pdf]

Supplementary materials

Table S1. Bins from the metagenome data of the sample NL01A and identification of Verrucomicrobiota

**Assembly tool**	MEGAHIT	MEGAHIT	MEGAHIT	MEGAHIT	MEGAHIT	MEGAHIT	MEGAHIT	MEGAHIT	MEGAHIT	MEGAHIT
**Alignment tool**	Bowtie2	Bowtie2	BWA	BWA	BWA	BWA	BWA	BWA	BWA	BWA
**Binning tool**	MetaBAT2	MaxBin2	MetaBAT2	MaxBin2	CONCOCT	MetaBAT2 MaxBin2	MaxBin2 CONCOCT	MetaBAT2 CONCOCT	MetaBAT2 MaxBin2 CONCOCT	Metawrap (re-assembly)
**No. of bins**	10	11	12	11	30 (12)	14	14	13	15	15
**Bin no. of Verrucomicrobiota**	Bin 9 (UID2982)	Bin 3 (UID3055)	Bin 8 (UID2982)	Bin 3 (UID2982)	Bin 3 (UID2982)	Refined Bin 10 (UID2982)	Refined Bin 10 (UID2982)	Refined Bin 9 (UID2982)	Refined Bin 10 (UID2982)	Refined Bin 10.orig (UID2982)
Completeness (%)	97.97	90.7	96.62	98.65	98.65	96.62	98.65	96.62	96.62	96.62
Contamination (%)	1.69	1.69	1.69	1.69	1.69	1.69	1.69	1.69	1.69	1.69
Genome size (bp)	2,139,525	2,240,973	2,119,528	2,240,973	2,269,719	2,119,528	2,233,703	2,119,528	2,119,528	2,119,528
GC contents (%)	40.6	40.9	40.6	40.9	41.1	40.6	40.9	40.6	40.6	40.6
Predicted genes	2,024	2,147	2,009	2,147	2,180	2,009	2,135	2,009	2,009	2,009

Table S1. Bins from the metagenome data of the sample NL01A and identification of Verrucomicrobiota (continued)

**Assembly tool**	Metaspades	Metaspades	Metaspades	Metaspades	Metaspades	Metaspades	Metaspades	Metaspades
**Alignment tool**	BWA	BWA	BWA	BWA	BWA	BWA	BWA	BWA
**Binning tool**	MetaBAT2	MaxBin2	CONCOCT	MetaBAT2<br>MaxBin2	MaxBin2<br>CONCOCT	MetaBAT2<br>CONCOCT	MetaBAT2<br>MaxBin2<br>CONCOCT	Metawrap<br>(re-assembly)
**No. of bins**	11	12	43	15	12	17	14	14
**Bin no. of Verrucomicrobiota**	Bin9<br>(UID 2982)	Bin2<br>(UID 2982)	Bin22<br>(UID 2982)	Refined<br>Bin 11<br>(UID 2982)	Refined<br>Bin 4<br>(UID 2982)	Refined<br>Bin 11<br>(UID 2982)	Refined<br>Bin 11<br>(UID 2982)	Refined Bin 11.<br>permissive<br>(UID 2982)
Completeness (%)	92.57	98.65	98.65	92.57	98.65	92.57	92.57	97.30
Contamination (%)	1.69	1.69	1.69	1.69	1.69	1.69	1.69	3.45
Genome size (bp)	2,207,440	2,467,065	2,340,822	2,207,440	2,340,822	2,207,440	2,207,440	2,689,209
GC contents (%)	41.34	41.26	41.43	41.34	41.43	41.34	41.34	42.10
Predicted genes	2,097	2,392	2,233	2,097	2,233	2,097	2,097	2,777

Table S2. Best bin candidates for identifying Verrucomicrobiota from the sample NL01A after binning, bin refinement, and re-assembly

	YNP I	YNP II	YNP III	YNP IV	YNP V
Assembly tool	MEGAHIT	MEGAHIT	MEGAHIT	Metaspades	Metaspades
Alignment tool	Bowtie2	BWA	BWA	BWA	BWA
Binning tool	MetaBAT2	MaxBin2	CONCOCT	MaxBin2	Metawrap (Re-assembly)
No. of bins	10	11	30	12	14
Bin no. of Verrucomicrobiota	Bin 9 (UID2982)	Bin 3 (UID2982)	Bin 3 (UID2982)	Bin2 (UID 2982)	Refined bin 11. permissive (UID 2982)
Completeness	97.97%	98.65%	98.65%	98.65%	97.30%
Contamination	1.69%	1.69%	1.69%	1.69%	3.45%
Genome size	2,139,525 bp	2,240,973 bp	2,269,719 bp	2,467,065 bp	2,689,209 bp
GC contents	40.6%	40.9%	41.1 %	41.26%	42.10%
Predicted genes	2,024	2,147	2,180	2,392	2,777
No. of *pmo* genes	6	8	8	10	10
Mapping reads					
M. fumariolicum SolV	39,667,086 (91.19%)	40,433,082 (88.85%)	40,521,352 (88.14%)	40,821,695 (82.70%)	40,603,025 (63.82%)
M. infernorum V4	6,483,503 (15.03%)	6,560,496 (14.54%)	6,575,906 (14.43%)	6,599,092 (13.48%)	6,598,174 (10.44%)
M. kamchatkense Kam1	38,536,935 (89.04%)	39,538,625 (87.30%)	39,685,000 (86.74%)	39,912,603 (81.22%)	39,709,491 (62.63%)

Mapping was conducted by BWA against the reference genome of *M. fumariolicum* SolV, *M. infernorum* V4, and *M. kamchatkense* Kam1.

Table S3. Genes associated with methane oxidation, CO_2 fixation, and nitrogen metabolism in strains YNP IV, SolV, V4, Kam1 and Yel

**Annotation**	**Gene**	**YNP IV**	**Identities (%)**			
**I. Methane oxidation**			**SolV**	**V4**	**Kam1**	**Yel**
***pmo* clusters**						
Methane/ammonia monooxygenase subunit C	*pmoC-amoC*	PPMHHGHK_00155	61.00	61.00	97.00	100
Methane/ammonia monooxygenase subunit A	*pmoA-amoA*	PPMHHGHK_00156	64.00	67.00	67.00	100
Methane/ammonia monooxygenase subunit C	*pmoC-amoC*	PPMHHGHK_00180	97.45	77.18	62.44	99.27
Methane/ammonia monooxygenase subunit B	*pmoB-amoB*	PPMHHGHK_00186	50.00	48.57	50.71	98.57
Methane/ammonia monooxygenase subunit B	*pmoB-amoB*	PPMHHGHK_00187	92.86	80.36	94.64	82.14
Methane/ammonia monooxygenase subunit C	*pmoC-amoC*	PPMHHGHK_00188	74.14	93.85	59.62	73.19
Methane/ammonia monooxygenase subunit A	*pmoA-amoA*	PPMHHGHK_00189	84.08	84.08	83.67	100
Methane/ammonia monooxygenase subunit B	*pmoB-amoB*	PPMHHGHK_00190	-	92.38	97.38	99.30
Methane/ammonia monooxygenase subunit C	*pmoC-amoC*	PPMHHGHK_00194	72.62	95.49	59.62	71.74
Methane/ammonia monooxygenase subunit C	*pmoC-amoC*	PPMHHGHK_00467	43.13	41.67	42.65	99.65
Methane/ammonia monooxygenase subunit A	*pmoA-amoA*	PPMHHGHK_00468	41.60	45.50	45.97	100
Methane/ammonia monooxygenase subunit B	*pmoB-amoB*	PPMHHGHK_00469	-	82.65	47.41	100
**Methanol dehydrogenase**						
Lanthanide-dependent methanol dehydrogenase	*xoxF*	PPMHHGHK_01377	97.71	95.58	97.66	100
XoxJ protein	*xoxJ*	PPMHHGHK_01378	96.90	79.56	96.21	100

Table S3. Genes associated with methane oxidation, CO_2 fixation, and nitrogen metabolism in strains YNP IV, SolV, V4, Kam1 and Yel (continued)

Annotation	Gene	YNP IV	Identities (%)			
**I. Methane oxidation (continued)**			**SolV**	**V4**	**Kam1**	**Yel**
**Methylamine dehydrogenase**						
Methylamine dehydrogenase light chain	*mauA*	PPMHHGHK_02191	93.33	72.41	94.44	100
Methylamine dehydrogenase heavy chain	*mauB*	PPMHHGHK_02194	93.35	65.61	94.56	100
**Formate dehydrogenase**						
Formate dehydrogenase	FDH	PPMHHGHK_00501	93.72	93.95	93.15	99.5
Formate dehydrogenase subunit gamma	*fdoI, fdsG*	PPMHHGHK_00570	96.03	81.33	96.05	99.34
Formate dehydrogenase iron-sulfur subunit	*fdoH, fdsB*	PPMHHGHK_00571	98.08	83.69	97.24	100
Formate dehydrogenase major subunit	*fdoG, fdhF, fdwA*	PPMHHGHK_00572	98.64	88.57	98.02	100
Formate dehydrogenase subunit delta	*fdsD*	PPMHHGHK_00573	93.67	72.00	97.47	100

Table S3. Genes associated with methane oxidation, CO_2 fixation, and nitrogen metabolism in strains YNP IV, SolV, V4, Kam1 and Yel (continued)

**Annotation**	**Gene**	**YNP IV**	**Identities (%)**			
**II. CO_2 fixation**			**SolV**	**V4**	**Kam1**	**Yel**
Carbonic anhydrase	*cynT, can*	PPMHHGHK_01486	96.91	79.27	97.94	100
Ribulose-bisphosphate carboxylase large chain	*rbcL, cbbL*	PPMHHGHK_00540	98.77	97.52	98.97	99.79
Ribulose-bisphosphate carboxylase small chain	*rbcS, cbbS*	PPMHHGHK_00541	96.38	92.03	91.30	100
Phosphoglycerate kinase	*pgk*	PPMHHGHK_01418	98.27	80.94	98.51	99.50
Glyceraldehyde 3-phosphate dehydrogenase	GAPDH, *gapA*	PPMHHGHK_01419	97.98	89.05	97.12	99.71
Fructose-bisphosphate aldolase, class II	*FBA, fbaA*	PPMHHGHK_01772	98.46	94.44	99.38	100
Fructose-1,6-bisphosphatase I / sedoheptulose-1,7-bisphosphatase	*fbp-SEBP*	PPMHHGHK_01330	97.75	87.14	97.47	100
Transketolase	*tktA, tktB*	PPMHHGHK_00544	97.94	82.87	96.57	100
Xylulose-5-phosphate/fructose-6-phosphate phosphoketolase	*xfp, xpk*	PPMHHGHK_00848	97.72	86.04	96.83	100
Phosphoribulokinase	PRK, *prkB*	PPMHHGHK_00543	99.70	96.71	98.51	100
Phosphoribulokinase	PRK, *prkB*	PPMHHGHK_01329	-	96.91	-	100
Ribulose-phosphate 3-epimerase	*rpe,* RPE	PPMHHGHK_01252	97.37	86.55	97.81	100
Ribulose-phosphate 3-epimerase	*rpe,* RPE	PPMHHGHK_02271	97.78	82.22	99.11	100
Fructose-bisphosphate aldolase, class II	FBA, *fbaA*	PPMHHGHK_01772	98.46	94.44	99.38	100
Fructose-1,6-bisphosphatase II	*glpX*	PPMHHGHK_00449	99.46	92.10	99.45	100
Ribose 5-phosphate isomerase B	*rpiB*	PPMHHGHK_00710	97.96	92.47	99.32	100
Triosephosphate isomerase (TIM)	TPI, *tpiA*	PPMHHGHK_01417	96.86	80.08	95.69	100
Glucose-6-phosphate isomerase	GPI, *pgi*	PPMHHGHK_01401	97.12	80.70	97.26	100
Glucose-6-phosphate 1-dehydrogenase	G6PD, *zwf*	PPMHHGHK_00196	97.70	89.13	98.47	100
Glucose-6-phosphate 1-dehydrogenase	G6PD, *zwf*	PPMHHGHK_00647	98.61	89.46	98.78	99.80
6-phosphogluconate dehydrogenase	PGD, *gnd, gntZ*	PPMHHGHK_00709	94.27	73.46	94.06	99.79
Transaldolase	*talA, talB*	PPMHHGHK_00539	92.62	68.96	92.84	100
Transaldolase	*talA, talB*	PPMHHGHK_01059	97.78	78.67	95.84	99.72

Table S3. Genes associated with methane oxidation, CO_2 fixation, and nitrogen metabolism in strains YNP IV, SolV, V4, Kam1 and Yel (continued)

**Annotation**	**Gene**	**YNP IV**	**Identities (%)**			
**III. Nitrogen metabolism**			**SolV**	**V4**	**Kam1**	**Yel**
**Nitrogen fixation**						
Pyruvate-ferredoxin/flavodoxinoxidoreductase	*por, nifJ*	PPMHHGHK_00040	96.36	73.67	94.42	100
Ferredoxin like protein	*fixX*	PPMHHGHK_00990	-	79.79	97.87	-
Electrontransferflavoprotein-quinoneoxidoreductase	*fixC*	PPMHHGHK_00991	96.30	81.48	97.69	100
Electron transfer flavoprotein alpha subunit	*fixB, etfA*	PPMHHGHK_00992	95.15	79.41	94.79	99.73
Electron transfer flavoprotein beta subunit	*fixA, etfB*	PPMHHGHK_00993	95.70	81.39	94.60	100
Nitrogenase-stabilizing/protective protein	*nifW*	PPMHHGHK_00994	96.69	65.83	97.52	100
Nitrogen fixation protein NifT	*nifT*	PPMHHGHK_00998	-	88.41	98.55	-
Iron-sulfur cluster insertion protein	*erpA*	PPMHHGHK_01000	91.73	81.58	93.23	99.17
Nif-specific regulatory protein	*nifA*	PPMHHGHK_01001	96.79	77.74	94.71	100
Nitrogen fixation protein NifQ	*nifQ*	PPMHHGHK_01004	93.65	61.70	89.22	-
Nitrogen fixation protein NifZ	*nifZ*	PPMHHGHK_01006	-	84.81	-	100
Nitrogen fixation protein NifB	*nifB*	PPMHHGHK_01011	98.47	90.23	98.28	99.81
Nitrogen fixation protein NifX	*nifX*	PPMHHGHK_01016	96.97	86.36	96.97	99.24
Nitrogenase molybdenum-iron protein NifN	*nifN*	PPMHHGHK_01017	97.51	79.47	96.42	99.78
Nitrogenase molybdenum-cofactor synthesis protein NifE	*nifE*	PPMHHGHK_01018	99.35	91.50	98.04	100
Nitrogenasemolybdenum-ironproteinbetachain	*nifK*	PPMHHGHK_01019	97.72	91.83	98.10	100
Nitrogenase molybdenum-iron protein alpha chain	*nifD*	PPMHHGHK_01020	98.57	93.48	98.17	99.80
Nitrogenase iron protein NifH	*nifH*	PPMHHGHK_01021 (319)	98.99	97.64	99.33	99.66

Table S3. Genes associated with methane oxidation, CO_2 fixation, and nitrogen metabolism in strains YNP IV, SolV, V4, Kam1 and Yel (continued)

**Annotation**	**Gene**	**YNP IV**	**Identities (%)**			
**III. Nitrogen metabolism (continued)**			**SolV**	**V4**	**Kam1**	**Yel**
HomocitratesynthaseNifV	*nifV*	PPMHHGHK_01022	93.25	74.27	93.78	100
Nitrogen fixation protein NifU and related proteins	*iscU, nifU*	PPMHHGHK_01763	95.00	63.31	92.14	100
**Nitrogen assimilation**						
Ammonium transporter, Amt family	*amt, AMT, MEP*	PPMHHGHK_01100	98.58	88.30	97.84	100
Nitrogen regulatory protein P-II 1	*glnB*	PPMHHGHK_00601	-	96.49	100	-
Nitrogen regulatory protein P-II 1	*glnB*	PPMHHGHK_01095	87.30	53.97	90.48	100
MFS transporter, NNP family, nitrate/nitrite transporter	*NRT, narK, nrtP, nasA*	PPMHHGHK_01086	94.00	67.21	91.51	99.54
Nitrite reductase (NADH) large subunit	*nirB*	PPMHHGHK_01084	95.33	69.37	94.34	99.75
Nitrite reductase (NADH) small subunit	*nirD*	PPMHHGHK_01085	91.45	52.34	91.45	98.99
Assimilatory nitrate reductase catalytic subunit	*nasA*	PPMHHGHK_01083	94.97	68.05	94.39	100
**Hydroxylamine detoxification**						
Hydroxylamine dehydrogenase	*hao*	PPMHHGHK_01994	94.83	72.54	94.40	100
Nitric oxide reductase subunit B	*norB*	PPMHHGHK_01443	90.18	80.91	98.66	100
Nitric oxide reductase subunit C	*norC*	PPMHHGHK_01444	90.48	80.09	95.93	100
**Nitrite reduction**						
Nitrite reductase (NO-forming)	*nirK*	PPMHHGHK_00871	97.86	88.06	-	100

Table S4. Number of antimicrobial resistant (AMR) gene families in the YNP IV genome assembly

AMR gene family	Number
Resistance-nodulation-cell division (RND) antibiotic efflux pump	34
ATP-binding cassette (ABC) antibiotic efflux pump	23
Major facilitator superfamily (MFS) antibiotic efflux pump	17
pmr phosphoethanolamine transferase	11
Antibiotic-resistant isoleucyl-tRNA synthetase (*ileS*)	4
Tetracycline-resistant ribosomal protection protein	4
Major facilitator superfamily (MFS) antibiotic efflux pump; resistance-nodulation-cell division (RND) antibiotic efflux pump	3
Elfamycin resistant EF-Tu	2
General Bacterial Porin with reduced permeability to beta-lactams	2
Intrinsic peptide antibiotic resistant Lps	2
Rifampin phosphotransferase	2
Sulfonamide resistant *sul*	2
vanH; glycopeptide resistance gene cluster	2
ABC-F ATP-binding cassette ribosomal protection protein	1
Antibiotic resistant *fabI*	1
Antibiotic resistant *fusA*	1
Antibiotic resistant *fusE*	1
Antibiotic resistant *kasA*	1
Antibiotic-resistant *murA* transferase	1
Antibiotic-resistant *ptsI* phosphotransferase	1
ATP-binding cassette (ABC) antibiotic efflux pump; major facilitator superfamily (MFS) antibiotic efflux pump	1
ATP-binding cassette (ABC) antibiotic efflux pump; major facilitator superfamily (MFS) antibiotic efflux pump; resistance-nodulation-cell division (RND) antibiotic efflux pump; General Bacterial Porin with reduced permeability to beta-lactams	1
Cfr 23S ribosomal RNA methyltransferase	1
Chloramphenicol acetyltransferase (CAT)	1
Erm 23S ribosomal RNA methyltransferase	1
Fluoroquinolone resistant *gyrB*	1
fosC phosphotransferase family	1
Glycopeptide resistance gene cluster; van ligase	1
Glycopeptide resistance gene cluster; *vanT*	1
kdpDE	1
Lipid A phosphatase	1
Methicillin resistant PBP2	1
Penicillin-binding protein mutations conferring resistance to beta-lactam antibiotics	1
Pyrazinamide resistant *pncA*	1
Resistance-nodulation-cell division (RND) antibiotic efflux pump; General Bacterial Porin with reduced permeability to beta-lactams	1
Rifampin monooxygenase	1
Rifamycin-resistant beta-subunit of RNA polymerase (*rpoB*)	1
Streptogramin vat acetyltransferase	1
Subclass B3 LRA beta-lactamase	1
Tetracycline inactivation enzyme	1
Undecaprenyl pyrophosphate related proteins	1
vanS; glycopeptide resistance gene cluster	1

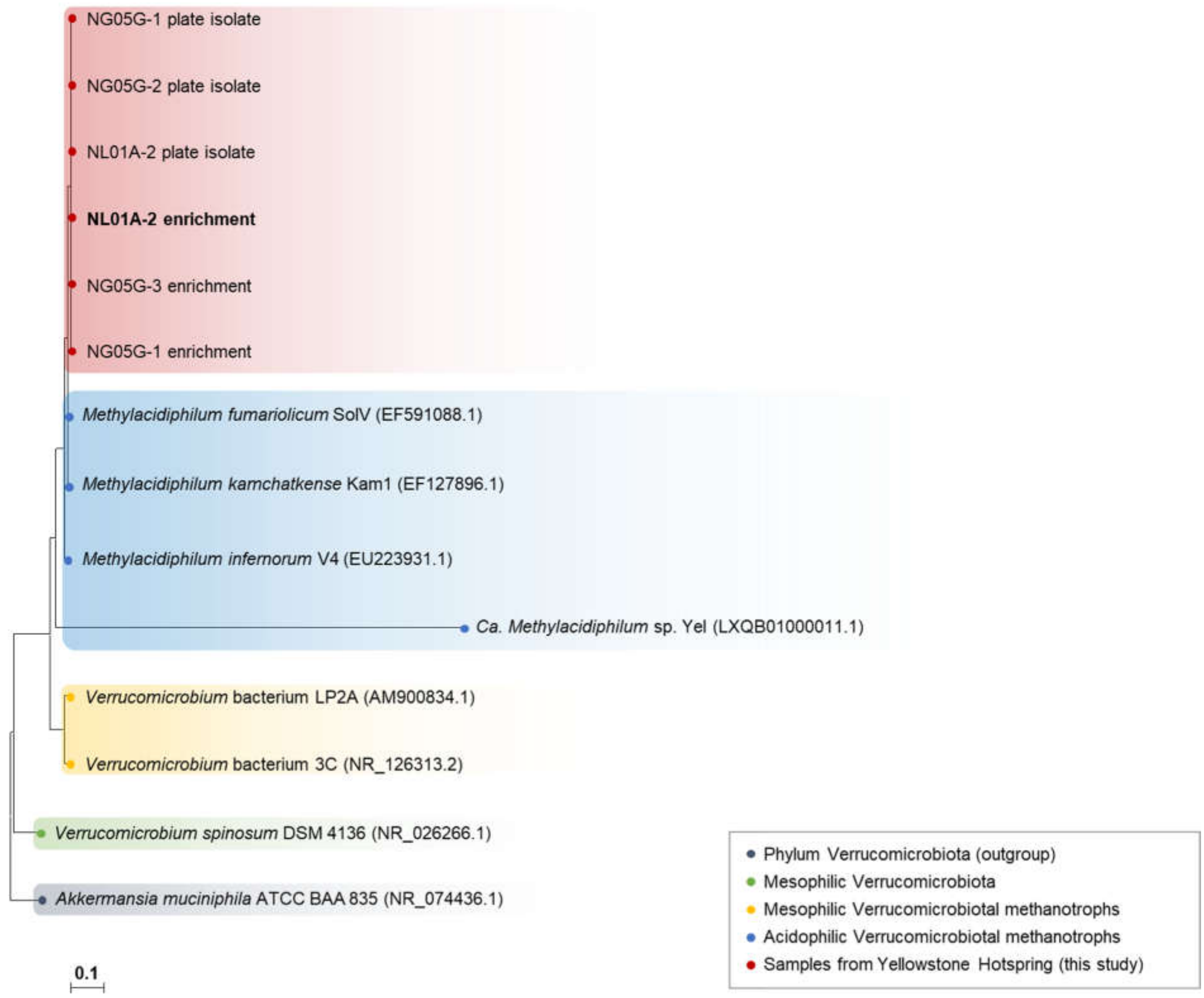

Figure S1. Phylogenetic analysis of Yellowstone hot spring samples and Verrucomicrobiota strains by the Maximum Likelihood tree using the Tamura-Nei model of the 16S rRNA gene sequences. Accession numbers for each reference bacteria are included in parentheses in the figure.

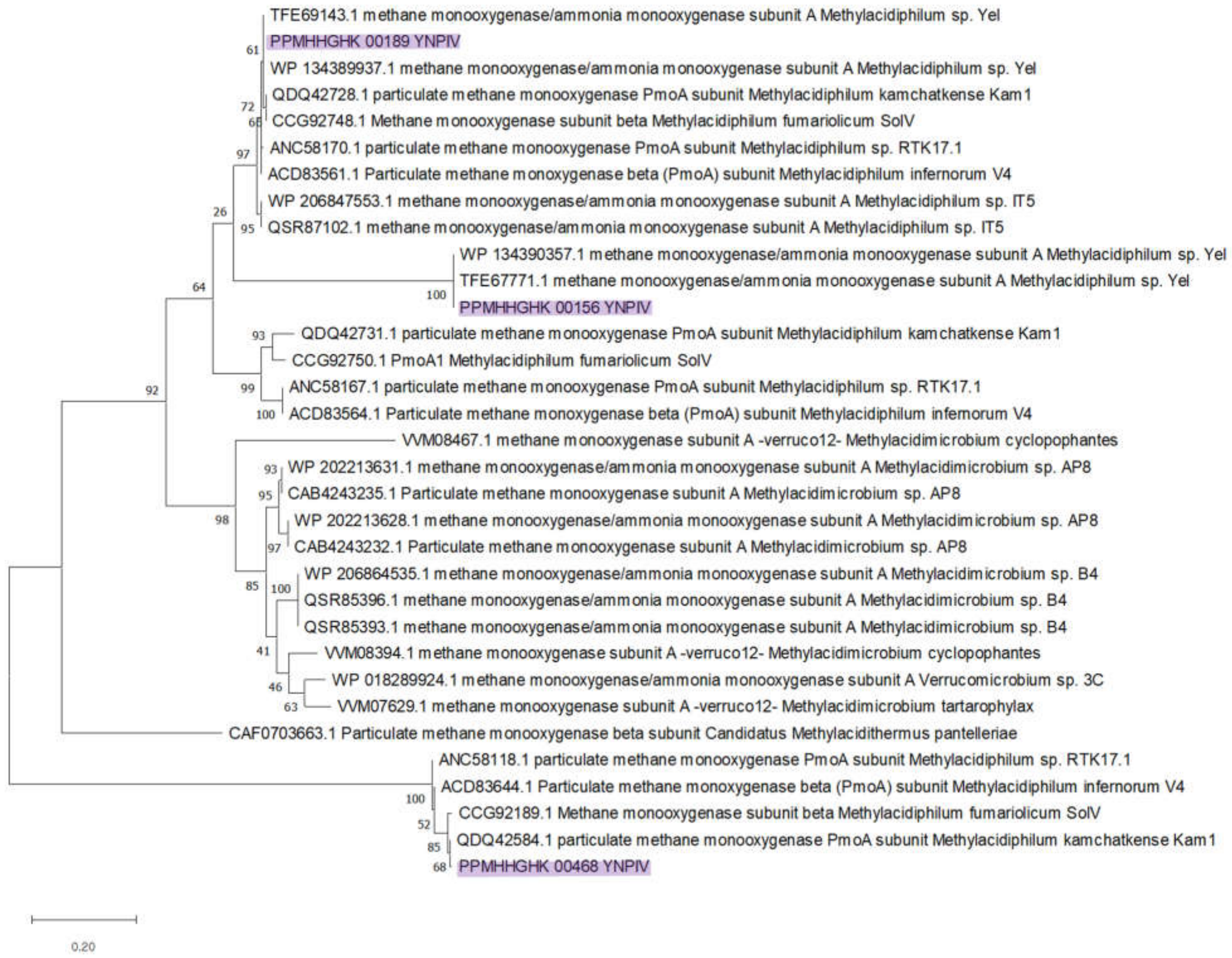

Figure S2. Phylogenetic analysis of the *pmoA* gene in YNP IV strain obtained using the Maximum Likelihood method. The tree is constructed from *pmoA* gene sequences of other references that have been obtained from the NCBI database. The percentage of trees where the associated taxa clustered together is presented next to the branches. Evolutionary analyses were conducted in MEGA-X.

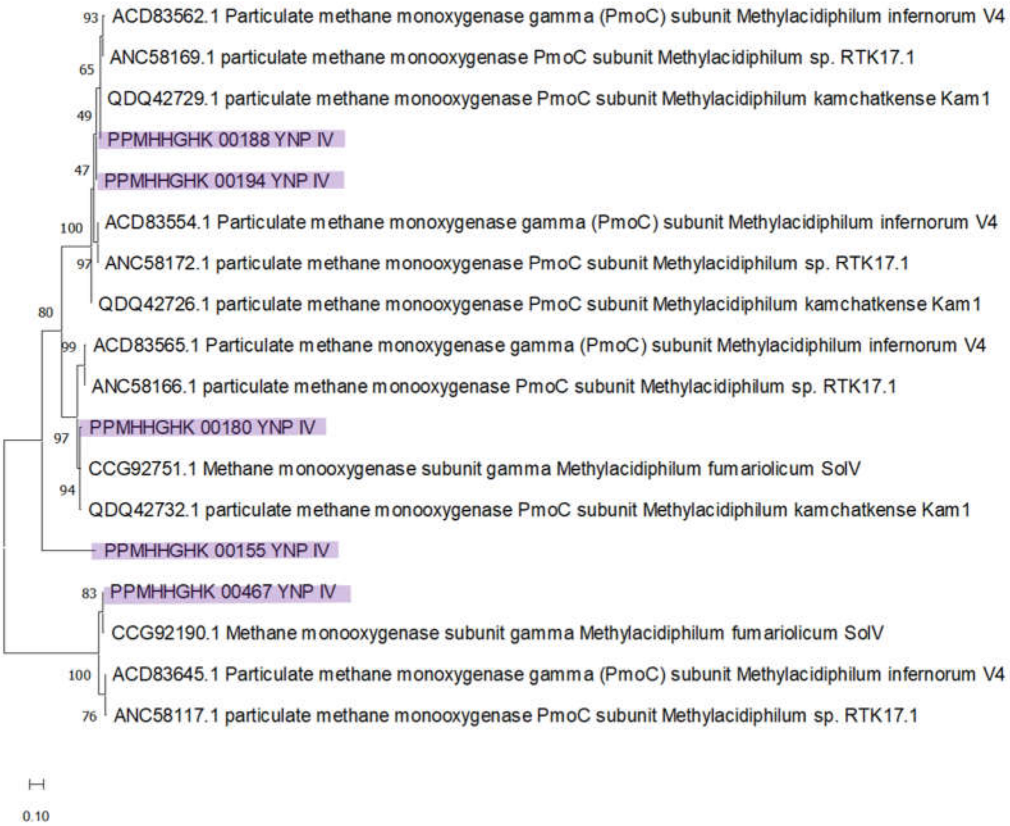

Figure S3. Phylogenetic analysis of the *pmoC* gene in YNP IV strain obtained using the Maximum Likelihood method. The tree is constructed from *pmoA* gene sequences of other references that have been obtained from the NCBI database. The percentage of trees where the associated taxa clustered together is presented next to the branches. Evolutionary analyses were conducted in MEGA-X.

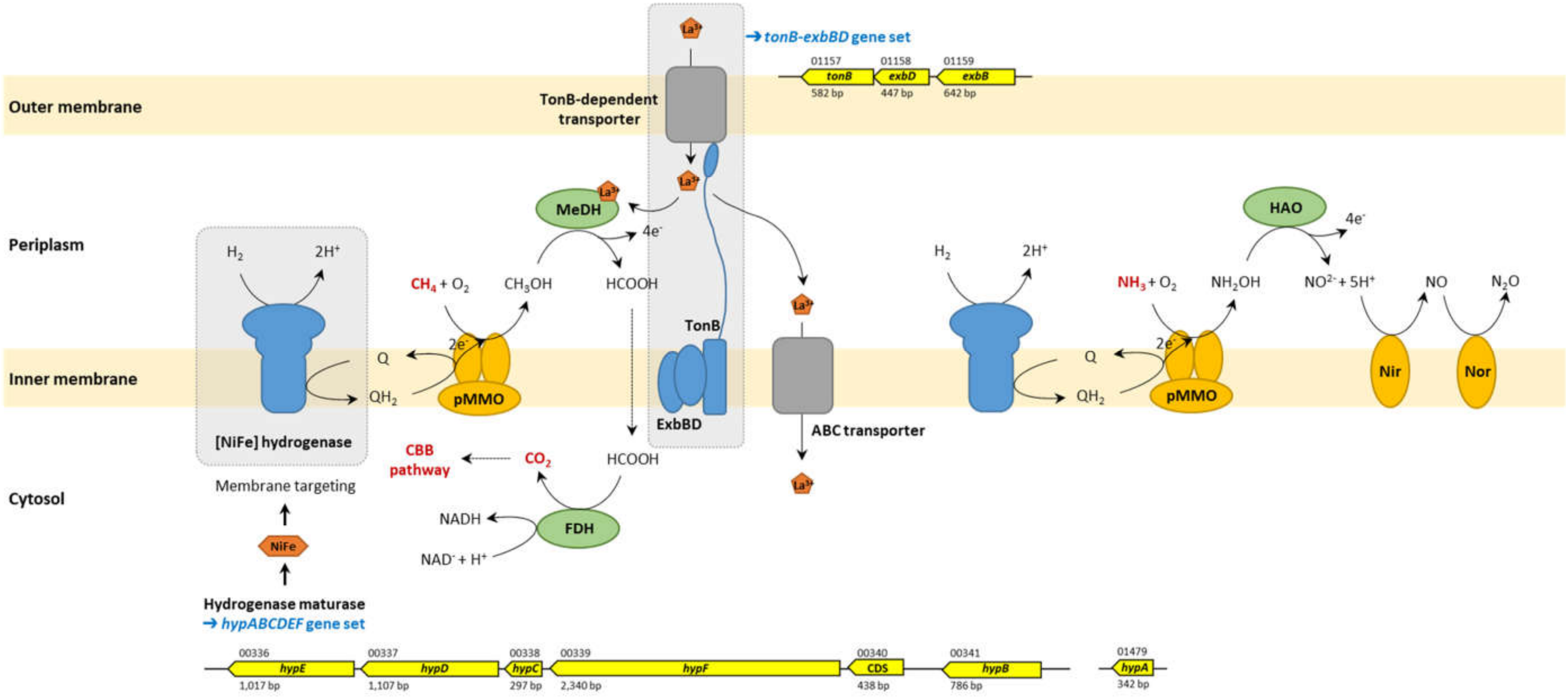

Figure S4. Hydrogenase genes related to methane and nitrogen metabolism and transportation of rare earth elements in the YNP IV. The numbers above and under the arrow are locus tag numbers without prefix (PPMHHGHK) and gene size.

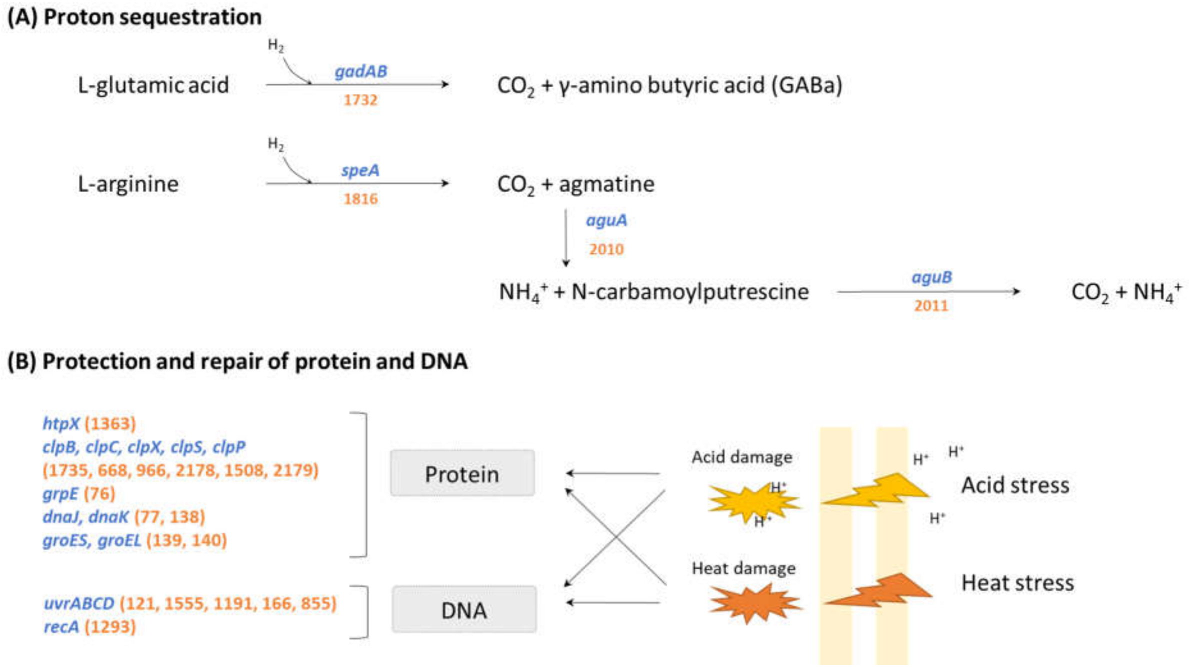

Figure S5. Acid and heat stress-associated genes in the YNP IV. (A) Proton sequestration. (B) Protection and repair of protein and DNA. Blue letters represent gene names and orange numbers indicate locus tag numbers without prefix (PPMHHGHK).

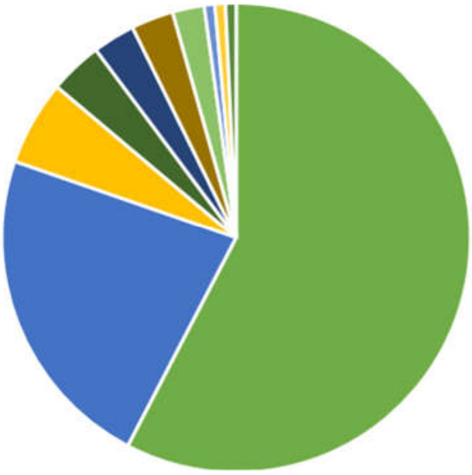

Resistance Mechanism	%
Antibiotic efflux	57.7
Antibiotic target alteration	22.6
Antibiotic inactivation	5.8
Antibiotic target protection	3.6
Reduced permeability to antibiotic	2.9
Antibiotic target alteration; antibiotic efflux	2.9
Antibiotic target replacement	2.2
Antibiotic target alteration; antibiotic efflux; reduced permeability to antibiotic	0.7
Antibiotic efflux; reduced permeability to antibiotic	0.7
Antibiotic target alteration; antibiotic target replacement	0.7

Figure S6. Pie graph showing the percentage of antimicrobial resistance mechanism related genes in the YNP IV genome assembly.
